# Impact of Education 4.0 among engineering students for learning English language

**DOI:** 10.1371/journal.pone.0261717

**Published:** 2022-02-02

**Authors:** V. Srivani, A. Hariharasudan, Nishad Nawaz, Sabina Ratajczak

**Affiliations:** 1 Faculty of English, Kalasalingam Academy of Research and Education, Anand Nagar, Krishnankoil, Tamil Nadu, India; 2 Department of Business Management, College of Business Administration, Kingdom University, Riffa, Bahrain; 3 Faculty of Applied Sciences, Department of Pedagogy, WSB University, Dąbrowa Górnicza, Poland; Rzeszow University of Technology: Politechnika Rzeszowska im Ignacego Lukasiewicza, POLAND

## Abstract

Education 4.0 is considered a significant technology for teaching and learning. The present study aims to explore the impact and the importance of Education 4.0 for improving English language learning in the perception of students in India, especially Hyderabad City. Moreover, Education 4.0 has bloomed as an important need to move along with the fast-growing education system of the world. But, it is a fact that many Indian students have complications and difficulties in learning English due to many reasons. The traditional methods of teaching are one of those reasons. Students learn only through the conventional methods, and they may find it boring and not effective. The education and mode of teaching have changed a lot, and it has attained a newer form of using the technology. Moreover, in the current era, where work from home and online teaching has become a new normal throughout the world, the technology used for teaching is inseparable. Hence, the authors are motivated to study the impact of Education 4.0 on improving English learning from Hyderabad, India. The methodology of the study has applied quantitative research. The study has employed pre-tested, close-ended questionnaires and post-assessment to gather data from the respondents to understand students’ performance in English language learning after implying Education 4.0. The five-point Likert scale has been used to analyse the collected data and to get mean values of responses. The total number of respondents is 145 students who pursue their engineering degrees in the select region. Among the collected data, most of them were from urban. The data obtained from the respondents proved that there is a direct correlation between the students’ perception and implementation of Education 4.0 in learning the English language. This study indicated that the respondents upheld Education 4.0 to improve English language learning in Hyderabad, India.

## Introduction

In the modern era, the use of Education 4.0 in learning is the utmost important method in learning. The approach of Education 4.0 is believed that it might promote innovative and smart behavior in education. Generally, Education 4.0 introduces technology-based strategies that promoting quality education. This can be described that students need not move behind lecture-based approaches, textbook materials and traditional mode of classrooms. Alternatively, students are encouraged to utilize the resources available on the Internet and get themselves registered in online courses, flipped lectures, video conferences, seminars and video calls to deliver course content [1]. Hence, Education 4.0 is encouraged in all educational colleges and universities to improve students’ English language learning. Students are considered the primary stakeholders in the educational world and are the promising beneficiaries in the educational ecosystem [2]. Education 4.0 technologies allow students to connect with a broad network and enhanced communication platforms with the stakeholders, parents, and teachers. The outcomes of the students learning process are directly related to the technologies involved in Education 4.0. The implementation of Education 4.0 benefits students’ learning process, which is more effective than the traditional approaches of learning. Specifically, Education 4.0 is emphasized in places where students’ learning is personalized. It can also be said that Education 4.0 drastically attracts or develops an interest in designing the curriculum for students learning. Education 4.0 technology attracts students because it creates a platform for more accessible videos and animations in the learning process, making students feel interested in learning [3,4]. This innovative technology creates easy access to lecture materials to the students, which significantly improves student learning [5]. In their turn, these opportunities are especially valuable considering the essential role of quality education among other constituents of the well-being of the youth and higher education universities [6,7]. As for the consequences of the whole country, it is proved that education positively influences economic growth and its quality [8–10]. Such results of efficient learning are significant for developing countries and regions [11–13]. Particularly, the interaction to the Internet access in schools, fixed broadband penetration and the latest available technologies affect product development in emerging markets [14,15]. Besides, in this Covid-19 situation, the education sector has been affected a lot. Though Education 4.0 was there before the pandemic, it got famous during the Covid-19 situation and improved the students’ self-centred learning capacity. The adoption of Education 4.0 helps higher education institutions to avoid any lacking in learning. The role of Education 4.0 is unimaginable, especially during the Covid-19 situation [16].

Education 4.0 creates a motivational learning process, making easy access to information and innovative technologies [17,18]. Puncreobutr critically investigated the need for Education 4.0 and described that this technology focuses explicitly on the students’ learning process benefiting the students in skill development and innovative technology implementation [19]. It significantly makes a clear view that students have enhanced access to learning information and modern technologies. The increasing possibilities of access to useful information are obvious due to the current ICT development [20–24]. Additionally, it is mandatory to promote Education 4.0 to the current generation students in classroom lectures. Despite promoting Education 4.0 to the students’ zone, it is necessary to adopt certain characteristics, as mentioned by Sadiyoko [25]. Sadiyoko suggests the following things concerning Education 4.0.

- Easy access of materials in all places and anytime
- Analyze the pieces of information based on the needs of the students
- Create a comfortable zone for delivering information
- Reflect peers and mentors
- Share learning information for answering specific “where” and “why” questions
- Symbolize the practical approach of students’ learning process
- Focus on projects and model-based learning concepts
- Describe students’ ownership where students’ participation is encouraged
- Focus on the process of evaluation of students’ learning outcomes

Considering students’ characteristics, it is promised to motivate Education 4.0 in learning the English language. The application and adoption of Education 4.0 in education are obligatory to keep pace with the fast-moving world. It is high time to start adopting Education 4.0 for education and learning the English language. Language learning reinforced by Education 4.0 would revolutionize education and aids in easy learning for students and teachers [26]. The South Indian students have some issues in learning English because of many reasons [27–30], which may also be predicted by utilizing innovative technologies [31]. One of the main reasons is the usage of the old teaching methods to teach a foreign language [32]. The second reason is the lack of foreign language practice and communication with teachers [33,34]. This can be overcome by the introduction of a technological component in teaching students. The main reason is that the English as a Foreign Language (EFL) can not practice or communicate the particular language with people around them. Hence, the learners can not ameliorate their language skills that lower the improvement of foreign language. The main objective of this study is to discover the perceptions of students on the impact of Education 4.0 on learning English language in South India. This study attempts to answer the following research question.

- What are the perceptions of students towards the impact of Education 4.0 on English language learning?

An overview of a detailed review of literature is proposed for a better understanding of the concept. The definitions and key concepts are presented in this section. It also gives an overview of the previous research related to our research problem and discusses the impact of Education 4.0 on improving English language learning. Also, it provides an overview of the earlier studies related to the literature that has discussed the effect of Education 4.0. Education 4.0 can be described as the device that makes intelligent decisions autonomously. Education 4.0 is a field that makes use of modern technologies in facilitating learning.

Ahmed-Ali conducted a study (2020) that investigates the effectiveness of Education 4.0 application in developing students’ oral language skills in EFL Egyptian primary school [35]. The study was conducted at an elementary school in Egypt. 40 students from sixth-year primary schools with poor English levels were selected. The study aimed at identifying the impact of Education 4.0 application to enhance listening and speaking skills in learning the English language. This quantitative research was conducted by collecting the data by a pre-test and a post-test. The participants were divided into two groups by the researchers. One group was trained by using the Google Assistant application for speaking skills, where the participants communicated through speaking with a robot and then listened to the robot’s response. On the other hand, another control group was taught by using the traditional method. From the pre-test and post-test, it is evident that Education 4.0 positively impacts improving listening and speaking skills in students of the experimental group. Moreover, it is observed that students were active in improving their listening and speaking skills, and they became more confident and professionals in using these skills. The researcher ensured that Education 4.0 made learning more accessible, effective and interesting. Furthermore, Education 4.0 crafted a realistic interactive platform for learning languages.

A study has been conducted on the Education 4.0 chatbots (a bot designed to converse with human beings) to improve English grammar skills in Korea [36]. The study’s objective was to find out the impact of the Education 4.0 chatbot in improving foreign language learning of university learners. It was quantitative research. The study was conducted among 70 students from a Korean University whose language proficiency was in different levels, such as beginners, intermediate and advanced. The study population was divided into two groups; one group was taught grammar using chat with Replika App, while the other group was taught by talking with their partner. It was noticed that both groups showed improvement, but the experimental group that used chatbots showed more significant improvement. Hence, the study concluded that Education 4.0 chatbots had an optimistic influence on learning a foreign language on Korean learners.

The study by Soliman (2016) in Bisha city of Saudi Arabia on utilizing a virtual learning environment was based on Education 4.0 to teach the English language to medical students [37]. The study stated that many earlier studies have described that many learners do not understand the medical English language material due to the inappropriate teaching methods used in that material. The study was quantitative research, and the data was collected from 29 faculty members of Bisha University. The study aimed at formulating a 3D Virtual Learning Environment based on Education 4.0. The result of the study had disclosed that most of the university’s faculty motivated the usage of Education 4.0 for the English language by learners. The researcher described that observing the genuineness of teaching English for medical students at Bisha University by incorporating a virtual learning environment based on Education 4.0 as the solution for the problems faced by the English language learners.

Whiteside and his group in 2017 studied improving the English language skills of native learners by using Education 4.0 [38]. In Tokyo, Japan, the study was conducted among 47 undergraduate native language learners with intermediate language proficiency. The study aimed at analyzing the impact of Education 4.0 on improving the native learners’ English language skills. Moreover, the researchers included coexistence with Education 4.0 for 21st-century skills in education. The study was mixed-method research. The samples were divided into two groups and eight subgroups. Google Home Mini was used to teach the first group to improve listening and speaking skills during the whole semester. Alexa was indulged in teaching the second group to improve listening comprehension and vocabulary skills throughout the semester. In addition, both groups were given some works like communication with AI speakers, watching subjects and writing summaries with 300-words, doing presentations, studying the theories of worldviews and the philosophies behind 21st-century skills, lastly, participating in discussions. The study revealed that both groups had improved English Language skills, and it is vivid that Education 4.0 incorporated with 21st-century skills aids in improving the English language skills of the learners.

The present study intends to explore students’ insights on Education 4.0 in improving English language learning in Hyderabad, India. Most of the previous studies were based on students or teachers only, while the present study focuses on the students’ perceptions. Additionally, the current research differs from the earlier works, as it significantly focuses on the perception of students and Telugu, being their mother tongue. Also, the previous studies followed a questionnaire for data collection, while the present study uses pre-test and post-test procedures and a detailed questionnaire to collect students’ data. However, this study focuses on students’ perception about using Education 4.0 on learning the English language. The discussions and results delivered in this research are different from the previous studies and the results. From the reviewed literature, it is evidenced that there is no such study has been conducted in this region concerning Education 4.0 for English Language Learning. It motivates the researchers to conduct research in the considered location. Further, it clearly shows the research gap of literature.

## Materials and methods

The current section brings out the methodology of the study and the research tools used to obtain solutions to the proposed research problems. Moreover, it also explains the respondents of the study and the procedure of data collection.

Based on the information obtained from the previous studies, the investigator has formulated the hypotheses according to the need of the present study as,

H_0_: There is no significant correlation between the perception of students on the implementation of Education 4.0 and the impact on English language learning.
H_1_: There is a significant correlation between the perception of students on the implementation of Education 4.0 and the impact on English language learning.

This is quantitative research. The data was collected using a close-ended, pre-tested questionnaire. The researchers or investigators designed hypotheses to define the significant parameters and variables and evaluated the obtained results.

As the present study is purely based on a quantitative approach, researchers proposed hypotheses for evaluating the students’ outcomes considering both the dependent and independent variables.

The present study was conducted to analyse the need of first-year students pursuing Engineering Colleges to learn the English language. After a detailed survey based on the literature studies, the researcher prepared materials as per the students’ needs in the present study. Opinions from experts were collected for validating the study materials and research instruments. Significant appreciation and positive comments were received from the expert members for the designed study materials. The study materials were prepared using Education 4.0 analysing the need of the students in concern with learning the English language.

Initially, the learning individuals were asked to attend the pre-test analysis and respond to the designed questionnaire to measure the students’ knowledge of the English language using Education 4.0. The answers were recorded and validated on the basis of students’ understanding of concepts. Next, the researchers utilize many Education 4.0 approaches for English language learning. The researchers use Facebook group discussions, Blog chats, WhatsApp discussions, YouTube and several mobile applications for training students in English language learning. Finally, a post-assessment analysis was performed along with a questionnaire. The pattern of post-assessment was followed as similar to the pre-assessment. The student individuals were asked to share their experience and difficulties in English language learning through the modern approach of Education 4.0 with detailed questionnaires and assessments. The score values of both the assessments and questionnaire were analysed to evaluate the effectiveness of Education 4.0 approaches in English language learning.

The questionnaires were framed for collecting the students’ data in both pre-assessments and post-assessments. The assessments were conducted in two modes (before and after) to evaluate the students’ reading, writing, vocabulary, speaking and grammatical skills. The students selected for this present study were encouraged to participate in pre- and post-assessments. The assessment pattern comprised of 6 sections: Grammar as Section I, Vocabulary as Section II, Reading as Section III, Listening as Section IV, Speaking as Section V and Writing as Section VI. Assessments conducted after the session help the researchers evaluate the students’ performance in English language learning after introducing Education 4.0 technology. The assessments were prepared in multiple choice-based questions format comprising 4 questions in each Sections I-IV with a weightage of 1 mark each. Section V and VI were evaluated based on the suggestions received from the International Standards of Reference for Languages with a weightage of 4 marks in each section. The selected participants were provided marks out of 24, containing 4 marks from each section.

Additionally, the participants selected in the present study attended a survey in the form of a questionnaire along with the assessments. Totally, two questionnaires were prepared and utilized for the survey along with the pre- and post-assessment. The survey contains eleven questions formulated in the English language for evaluating the students’ perception in English language learning after implementing Education 4.0 technology using five-point Likert scale analysis comprising strongly agree as level 1, agree as level 2, neutral as level 3, disagree as level 4 and strongly disagree as level 5. These questions provide a detailed report evaluating the student’s comfortabilities and difficulties while incorporating Education 4.0 for English language learning.

The researchers planned systematically conducting guidance sessions on the utilization of Education 4.0 strategies (Facebook, WhatsApp, YouTube channels, Blogs and several Mobile applications) in English language learning. The researchers constructed WhatsApp groups separately for boys and girls for each college, enabling effective communication with the selected learners. Also, the students were provided time to discuss with the mentors and researchers to solve their doubts. The researchers encouraged the students to listen to pre-recorded voice or group chats to assign works to the students, which motivated the students to use the Education 4.0 technology to learn English effectively.The participants selected in the present study are pursuing their education in six different engineering colleges situated in Hyderabad, India, implementing Education 4.0. The names of six engineering colleges are Vasavi College of Engineering, CMR Engineering College, MLR Institute of Technology, Holy Mary Institute of Technology and Science, Chaitanya Bharathi Institute of Technology and Siddhartha Institute of Technology and Sciences. Overall, six colleges were covered, and 145 students were selected for the current study. It was focused on selecting first-year engineering students from various disciplines, accounting for 120 male candidates and 25 female candidates. Also, colleges’ selection was based on the regional environment covering 45 rural students and 100 urban students. The reason for more female respondents is that most of the students are male in the targeted institutions. Consequently, the same has reflected in the data collection also. In addition to that, the targeted institutions are situated in an urban location, which is why most of the respondents are also from urban.

The data were collected from the informants (Engineering Students) from 1 February 2021 to 29 July 2021. Considering the primary step, the pre-assessment and post-assessment procedures were conducted for the selected participants to analyze the students’ performance in English language learning using Education 4.0. Also, two questionnaires were developed for students. The respondents were given a small introduction about the importance of Education 4.0 in improving English language learning. In addition, some videos on the impact of Education 4.0 in learning languages were included. The questionnaires were shared online. Then, the respondents were asked to respond to the questionnaires to analyze the comfortability and difficulties in using Education 4.0 for learning the English language in the regions of Hyderabad. Once the respondents had completed, the responses were quantitatively analyzed and discussed in detail.

The prime aim of this study was to examine the perceptions of students on the impact of using Education 4.0 on the effective learning of the English language. The data collected for the pre and post-test were analyzed to test the student’s outcome after implementing Education 4.0 for learning the English language. Also, the results gathered from questionnaires were collected, presented and discussed in this section. The researcher designed the questionnaires to answer the research question, which was: “What are the perceptions of students towards the impact of using Education 4.0 on English language learning?”

It is revealed from Table 1 that the pre-assessment and post-assessment contain six different sections with a weightage of 4 marks for each section, anlaysing the students’ language skills. The questions asked in the Grammar, Listening, Reading and Vocabulary sections contain multiple choice-based patterns. The questions in the Speaking section contain a task where students have to record their voices based on the random topic displayed on the Mobile screen. Next, participants will be assigned to write nearly 100–200 words related to the given topic in the writing section. These two sections were evaluated based on the International Standard of References for Languages.

**Table 1:** Students performance in English language.

Section	Students’ Performance	%
Grammar	62	42.76
Vocabulary	129	88.97
Reading	53	36.55
Listening	71	48.97
Speaking	121	83.45
Writing	135	93.10

The results obtained from the chart (S1 Fig) indicate a significant improvement in the students’ performance in English language learning while comparing both the pre- and post-assessment outcomes. The assessments contained six different sections like Grammar, Reading, Writing, Vocabulary, Speaking and Listening. After the implementation of Education 4.0, there is a drastic improvement in the students’ language skills. Considering the Grammatical section, 62 students (42.76%) were improved in the post-assessment compared to the pre-assessments. Considering the Vocabulary section, 129 students (88.97%) was found to improve in the post-assessment compared to the pre-assessment. Under the Reading category, 53 students (36.55%) improved in the post-assessment analysis. Next, analysing the Listening section, 71 students (48.97%) significantly improved in the post-assessment evaluation while compared with the pre-assessment results. Then, the Speaking section indicates that 121students (83.45%) significantly improved in the post-assessment analysis comparing to the pre-assessment results. Finally, analysis of the Writing section described that 135 students (93.1%) significantly improved in the post-assessment evaluation while compared with the pre-assessment results. Thus, based on the obtained results, it is clear that the implementation of Education 4.0 has made significant changes and improvements among the students in English language learning. Thus, it clearly depicts that the null hypothesis (H_0_) is rejected, and alternate hypothesis (H_1_) is accepted, which illustrates a correlation between the students’ perception and the implementation of Education 4.0 in English language learning.

A separate close-ended questionnaire for the students was prepared by the researchers, which comprised 11 statements. The main aim of this questionnaire was to examine the perception of the students towards Education 4.0. The results of the questionnaire are presented in Table 2.

**Table 2:** Response frequencies and mean for students’questionnaire.

Statements	Strongly Agree	Agree	Neutral	Disagree	Strongly Disagree	Mean Response
1. The use of Education 4.0 in education improves the educational environment for learning English language.	63	82	0	0	0	1.434
2. Education 4.0 is important to use these days in education to prepare language learners to acquire English language learning.	65	50	30	0	0	1.241
3. Education 4.0 contributes to language development faster.	55	62	28	0	0	1.186
4. Education 4.0 caters to all age groups’ needs and faster the ability in the language in English language learning.	52	68	25	0	0	1.186
5. Education 4.0 clarifies many points that the student cannot cover in their explanation.	65	49	31	0	0	1.234
6. Education 4.0 fulfils and complements all students’ language learning needs.	60	56	22	7	0	1.166
7. Education 4.0 enables students to obtain additional educational support for what the teacher does in English language classroom.	78	61	6	0	0	1.497
8. Learning through Education 4.0 will make learning a language less terrifying than learning it using the traditional way.	76	55	12	1	0	1.414
9. Education 4.0 changes the way how students acquire English language skills.	65	73	4	2	0	1.379
10. The teacher’s role will diminish when the student uses Education 4.0 to learn English.	61	70	10	3	0	1.297
11. The use of Education 4.0 affects the ability to communicate with the teacher.	58	77	6	1	0	1.303

Table 2 illustrates the responses to the questionnaire given to 145 students from Hyderabad. The students appeared with an overall positive attitude of using Education 4.0 in learning English. The first statement (1.434) is the most important statement of this study, which is used to elucidate the attitude of students towards learning using Education 4.0. Almost all the students are “agreed” and “strongly agreed” with the statement. In the second statement (1.241), most of the students “agreed” and “strongly agreed” that it is important to use Education 4.0 to acquire knowledge of the English language though few of them were neutral. In the third statement (1.186), most of the students “agreed” and “strongly agreed” that Education 4.0 facilitating faster language learning. It provides them with a contemporary source for learning the language by imitating as a real teacher, and some students were neutral because the presence of Education 4.0 enhanced the learning of language, but at the same time, it did not affect the quality of learning language if it is unused.

Using Education 4.0, the English language proficiency level of the student-respondents was determined and based on that, and the training was given accordingly to improve their language. For the fourth statement (1.186), most of the students “agreed” and “strongly agreed” that Education 4.0 furnishes the requirements of all age groups and hastens the English language learning ability. However, three students were neutral with the use of Education 4.0 in learning language because the users may enhance the educational process, and if it is not used, it will not affect the teacher’s role in learning the language within a short period. In the fifth statement (1.234), most of the students were “agreed” and “strongly agreed” that Education 4.0 aided language learning by simplifying the contents and clarifying many points that teachers cannot cover in the classroom lecture, since it helped students to be simulated and trained with proof-reader also had an explanation from an intelligent source other than the teacher. In addition, few of the students were neutral, saying that no one can completely take over and cover the role of the teachers.

The respondent’s statement“agreed” and “strongly agreed” that Education 4.0 accomplishes and complements all the students’ language learning needs as it could be used anytime and anywhere. In the sixth statement (1.166), few students were neutral; moreover, one student did not agree since the available technical advancement assistance can help meet the needs of language learning. In the seventh statement (1.497), most of the students “agreed” and “strongly agreed” that Education 4.0 supported students with extra educational assistance for what the teacher did in the English language classroom as it frolicked the part of the teacher in the simulation of language learning. However, some of the students were neutral to this statement.

In the eighth statement (1.414), most of the students “agreed” and “strongly agreed” that using Education 4.0 has made learning language less frightening than using the traditional way by creating a new method of teaching the students, which is less time consuming and more fun full. In addition, one student disagreed, and some students were neutral with this statement because they prefer studying more language skills by using traditional methods. In the ninth statement (1.379), most of the students “agreed” and “strongly agreed” that Education 4.0 changes the technique that students used to develop English language skills because it made students more eager, enthusiastic and passionate about communication. In the end, Education 4.0 is considered a device with which students are less nervous about making mistakes than in a classroom. Though one student was neutral, a few students disagreed, saying they may have complications because they may have difficulties dealing with the app\device and prefer a human teacher. The participants were attracted by the tenth statement (1.297), which was “When the student uses Education 4.0 to learn the English language, the teacher’s role will get reduced”. Most of the students “agreed” and “strongly agreed” because of the notorious development in the educational process because the teacher is not alone in training the students to learn the language. For this statement, few were neutral, and the others disagreed that the teachers’ role is crucial and did not diminish their role, though Education 4.0 is used. In the final statement (1.303) most of the students “agreed” and “strongly agreed” that Education 4.0 affected the capability of positive communication with the teacher. Few of the students were neutral for this statement; however, one student disagreed that Education 4.0 affected communication because the teacher plays a crucial role in training the students to learn the English Language.

## Discussion

The discoveries of this study are similar to some of the findings of the previous studies. It goes on par with the first Egyptian study by Ahmed-Ali (2020) on using Education 4.0 to develop primary school students’ oral language skills ensured that using Education 4.0 had shown significant improvement in students’ listening and speaking skills [35]. This study also supported the result of the study conducted by Kim (2020) on using Education 4.0 chatbots to improve English grammar skills, confirming that the usage of chatbots showed more noticeable improvement [39]. The present study corroborates with the previous work conducted by Murray et al. (2020), where recent technology is implemented in the process of learning the English language by the students [40]. Additionally, this study also aligned with the findings of the study directed by Soliman (2016) on the application of 3D virtual learning environment based on Education 4.0 for teaching the English language; the study authenticated that most of the university’s faculty motivated the usage of Education 4.0 for learning the English language [37]. Based on the data collected, it is observed that there is a correlation between the students’ perception and the usage of Education 4.0 approaches in language learning, improving the students’ language skills. A similar study corroborates with the survey analysis conducted by Yunus and team (2010) symbolizing high positive attitudes and critical thinking in the language learning platform through modern technologies [41]. Moreover, this study also supported the discovery of the previous study led by Whiteside about using Education 4.0 to enhance English language skills among native learners, revealing that Education 4.0 upgraded English language skills combined with 21st-century skills [38]. Overall, the results showed that students have positively used Education 4.0 to learn the English language effectively. It is clearly evidenced from the comparison of the previous results with results of the present study that the novelty of the study is highlighted from the results as there is no study concerning Education 4.0 for English Language Learning in the select region. Besides, the novelty of the study gives way to the educators to implement the suggestions given by the study as it improves the quality of self-centre learning.

## Conclusion

To conclude, this study has intended to inspect students’ perception of the application of Education 4.0 in improving English language learning. Combining Education 4.0 in language classrooms is significant to attain the contemporary methods of language learning. Especially from the results of this study, it can be concluded that students have shown a constructive positive attitude towards the usage of Education 4.0 in the effective learning of the English language. Further, it is proved that the usage of Education 4.0 has improved the English language efficiency of engineering students of Hyderabad, India.

The researchers would like to provide certain suggestions/recommendations after the completion of the research. Initially, the teachers who are desirable to teach the English language using Education 4.0 to the students are recommended to upgrade themselves to the modern strategies of Education 4.0, which might help them improve their knowledge and innovative skills. Also, students are recommended to follow Education 4.0 in English language learning. The teachers are recommended to participate in training programs to effectively utilise Education 4.0 approaches in teaching the English language to the students. Teachers should utilize this Education 4.0 technology and make the resources available to the students online for their learning. The teachers are strongly motivated to enrich their knowledge themselves with the latest developments in education and technology, since the future of education depends on developing an easy and suitable method for speeding up the educational process for learners, thereby ensuring the effective learning of the English Language. The recommendation to the researchers is that future researchers may conduct research on a qualitative basis because it may give additional inputs in this context. Though the present study has been conducted effectively, it also has some limitations. The participants included in the present study were mostly male respondents, and hence further studies need to be concentrated with equal participation of both male and female candidates. Another limitation in the present study is that participants are mostly selected from urban backgrounds compared to rural ones. These limitations must be resolved in future studies.

## Supporting information

S1 Fig Pre-test and post-test result analysis. (TIF)

S1 File Questionnaire. (PDF)

S2 File Overall data—master file. (XLSX)
