# Supplementary figures and images for: Impact of Education 4.0 among engineering students for learning English language
Source: PLoS One. 2022 Feb 2;17(2):e0261717. doi: 10.1371/journal.pone.0261717 (PMC8809588; doi:10.1371/journal.pone.0261717)

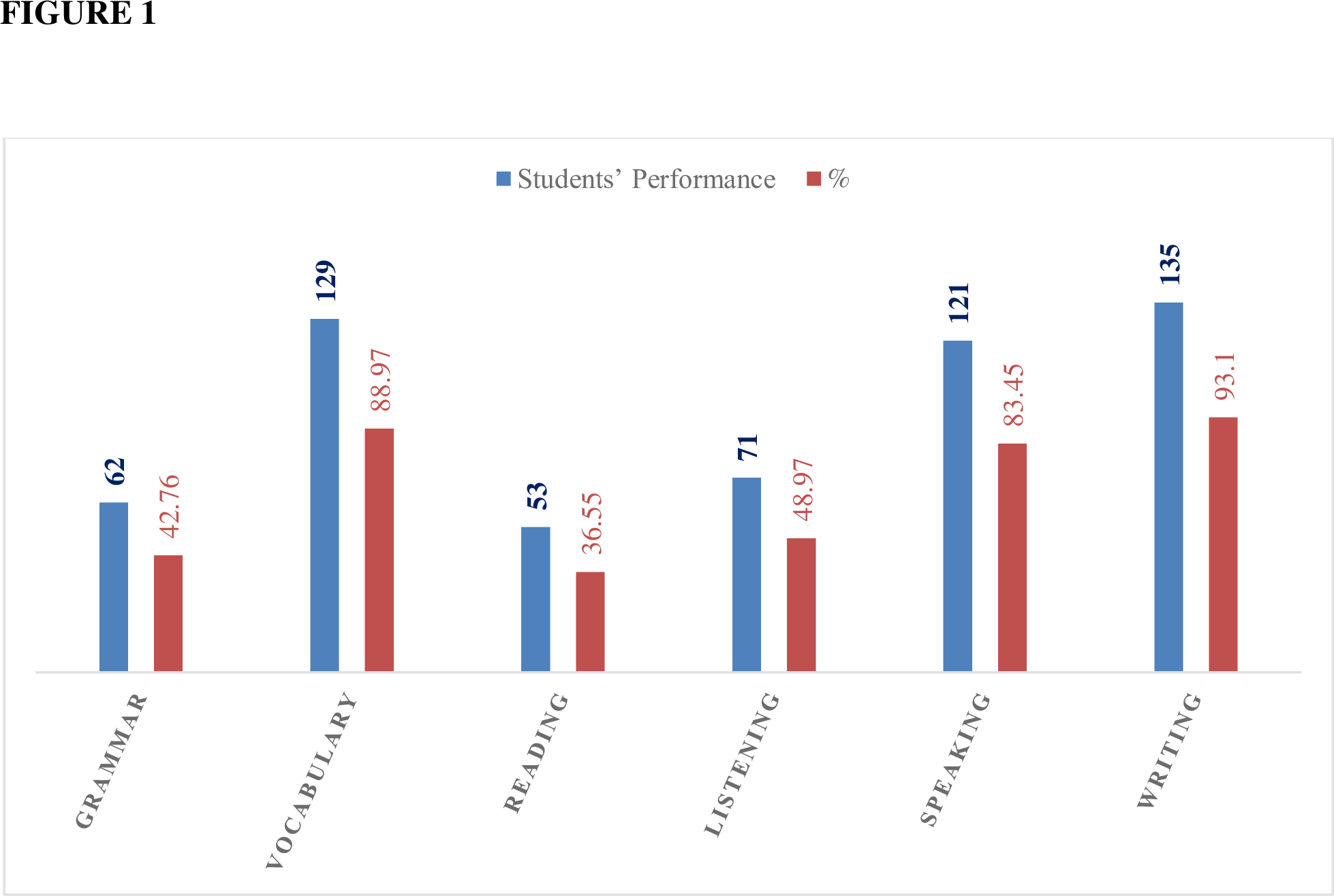

Supplement: S1 Fig — (TIF) [file pone.0261717.s001.tif]
