# Supplementary material for: Impact of Education 4.0 among engineering students for learning English language
Source: PLoS One. 2022 Feb 2;17(2):e0261717. doi: 10.1371/journal.pone.0261717 (PMC8809588; doi:10.1371/journal.pone.0261717)
Supplement: S1 File — (PDF) [file pone.0261717.s002.pdf]

Title: Impact of Education 4.0 among Engineering Students for Learning English Language

Questionnaire

S. No.	Statements	Strongly agree (2)	Agree (1)	Neutral (0)	Disagree (-1)	Strongly disagree (-2)
1	The use of Education 4.0 in education improves the educational environment for learning English language.					
2	Education 4.0 is important to use these days in education to prepare language learners to acquire English language learning.					
3	Education 4.0 contributes to language development faster.					
4	Education 4.0 caters the needs of all age group and faster the ability in language in English language learning.					
5	Education 4.0 clarifies many points that the student cannot cover in their explanation.					
6	Education 4.0 fulfils and complements all students' language learning needs.					
7	Education 4.0 enables students to obtain additional educational support for what the teacher does in English					

	language classroom.					
8	Learning through Education 4.0 will make learning language less terrifying than learning it using the traditional way.					
9	Education 4.0 changes the way how students acquire English language skills.					
10	The teacher's role will diminish when the student uses Education 4.0 to learn the English language.					
11	The use of Education 4.0 affects the ability to communicate with the teacher.					
